# Choice of HbA1c threshold for identifying individuals at high risk of type 2 diabetes and implications for diabetes prevention programmes: a cohort study

**DOI:** 10.1186/s12916-021-02054-w

**Published:** 2021-08-20

**Authors:** Lauren R. Rodgers, Anita V. Hill, John M. Dennis, Zoe Craig, Benedict May, Andrew T. Hattersley, Timothy J. McDonald, Rob C. Andrews, Angus Jones, Beverley M. Shields

**Affiliations:** 1 Institute of Health Research, University of Exeter Medical School, South Cloisters, St Lukes Campus, Exeter, EX1 2LU UK; 2 NIHR Exeter Clinical Research Facility, Royal Devon & Exeter NHS Foundation Trust & University of Exeter Medical School, Exeter, UK; 3 Institute of Biomedical and Clinical Science, College of Medicine and Health, University of Exeter, Exeter, EX2 5DW UK; 4 Clinical Trials Research Unit, Leeds Institute of Clinical Trials Research, University of Leeds, Leeds, West Yorkshire UK; 5 College of Mathematics Engineering and Physical Science, University of Exeter, Exeter, UK; 6 Department of Diabetes and Endocrinology, Royal Devon and Exeter NHS Foundation Trust, Exeter, UK; 7 Academic Department of Blood Sciences, Royal Devon and Exeter NHS Foundation Trust, Exeter, UK

**Keywords:** Non-insulin treated type 2 diabetes, Progression, Disease prevention, Cohort analysis, EXTEND, Pre-diabetes, HbA1c, Intermediate hyperglycaemia

## Abstract

**Background:**

Type 2 diabetes (T2D) is common and increasing in prevalence. It is possible to prevent or delay T2D using lifestyle intervention programmes. Entry to these programmes is usually determined by a measure of glycaemia in the ‘intermediate’ range. This paper investigated the relationship between HbA1c and future diabetes risk and determined the impact of varying thresholds to identify those at high risk of developing T2D.

**Methods:**

We studied 4227 participants without diabetes aged ≥ 40 years recruited to the Exeter 10,000 population cohort in South West England. HbA1c was measured at study recruitment with repeat HbA1c available as part of usual care. Absolute risk of developing diabetes within 5 years, defined by HbA1c ≥ 48 mmol/mol (6.5%), according to baseline HbA1c, was assessed by a flexible parametric survival model.

**Results:**

The overall absolute 5-year risk (95% CI) of developing T2D in the cohort was 4.2% (3.6, 4.8%). This rose to 7.1% (6.1, 8.2%) in the 56% (*n* = 2358/4224) of participants classified ‘high-risk’ with HbA1c ≥ 39 mmol/mol (5.7%; ADA criteria). Under IEC criteria, HbA1c ≥ 42 mmol/mol (6.0%), 22% (*n* = 929/4277) of the cohort was classified high-risk with 5-year risk 14.9% (12.6, 17.2%). Those with the highest HbA1c values (44–47 mmol/mol [6.2–6.4%]) had much higher 5-year risk, 26.4% (22.0, 30.5%) compared with 2.1% (1.5, 2.6%) for 39–41 mmol/mol (5.7–5.9%) and 7.0% (5.4, 8.6%) for 42–43 mmol/mol (6.0–6.1%). Changing the entry criterion to prevention programmes from 39 to 42 mmol/mol (5.7–6.0%) reduced the proportion classified high-risk by 61%, and increased the positive predictive value (PPV) from 5.8 to 12.4% with negligible impact on the negative predictive value (NPV), 99.6% to 99.1%. Increasing the threshold further, to 44 mmol/mol (6.2%), reduced those classified high-risk by 59%, and markedly increased the PPV from 12.4 to 23.2% and had little impact on the NPV (99.1% to 98.5%).

**Conclusions:**

A large proportion of people are identified as high-risk using current thresholds. Increasing the risk threshold markedly reduces the number of people that would be classified as high-risk and entered into prevention programmes, although this must be balanced against cases missed. Raising the entry threshold would allow limited intervention opportunities to be focused on those most likely to develop T2D.

**Supplementary Information:**

The online version contains supplementary material available at 10.1186/s12916-021-02054-w.

## Background

Type 2 diabetes (T2D) is both common and rapidly increasing in prevalence. In 2017, an estimated 425 million people were living with T2D, and this is projected to increase to 629 million by 2030 [1, 2]. As T2D is potentially preventable, the implementation of effective strategies to prevent the development of T2D is a priority [3]. Trials have shown that prevention programmes targeting those with the highest risk [4] may be effective in delaying the onset of T2D [5–9] for up to 15 years. While the relative merit of population vs targeted prevention is a subject of much debate [10, 11], many countries have targeted programmes. Within Europe, 13/22 countries have current national diabetes prevention programmes (DPPs) in place; two-thirds of these specifically target those at risk of developing diabetes [12]. Globally, examples of countries with DPPs include Australia, USA and China. While likely cost-effective, these programmes require substantial investment, costing between 0.13 and 0.2% of the annual healthcare budgets of the Netherlands, Germany and Australia [13, 14]. The UK has launched a national DPP which is expected to cost £105 million over a 5-year period [14–16].

A major issue for T2D prevention and the design of prevention programmes is defining the at-risk group to determine who should be enrolled. Entry to prevention programmes is usually based on a measure of glycaemia in the ‘intermediate’ range, often termed ‘pre-diabetes’ or ‘non-diabetic hyperglycaemia’. However, there is no consensus as to which test, and at what threshold, should be used to define pre-diabetes. The American Diabetes Association (ADA) defines the elevated risk range for pre-diabetes as an HbA1c 39–47 mmol/mol (5.7–6.4%), fasting plasma glucose (FPG) 5.6–6.9 mmol/L or oral glucose tolerance test (OGTT) 2-h glucose of 7.0–11.1 mmol/L. The International Expert Committee (IEC), World Health Organization (WHO) and many other countries use ranges of HbA1c 42–47 mmol/mol (6.0–6.4%), FPG 6.0–6.9 mmol/L or OGTT 7–11.1 mmol/L to indicate a high risk of developing diabetes [13]. Some guidelines, such as those in the UK [17], recommend a two-stage approach using a clinical risk score and HbA1c 42–47 mmol/mol (6.0–6.4%), where testing is targeted to specific groups at high-risk (e.g., Leicester Risk Score or Cambridge Risk Score) [18, 19].

In practice, opportune screening without a formal risk assessment is commonly undertaken, with HbA1c the most widely used measure due to ease of testing and convenience [20]. Systematic reviews have concluded this intermediate glycaemia predicts progression to T2D with varying accuracy depending on the definition and study population [6, 21–24]. A meta-analysis of studies examining the relationship between HbA1c and future diabetes identified 6 studies. They noted that progression rates using HbA1c ranges of 6.0–6.4% were similar to those using FPG ranges 5.6–6.9 mmol/L and that further studies on the predictive performance of HbA1c on progression to T2D are required [22]. There has also been criticism of the number of participants deemed to be at risk of developing T2D using these tests. For example, HbA1c testing using the IEC/WHO definitions of risk of developing diabetes (HbA1c 42–47 mmol/mol [6.0–6.4%]) 19% of UK adults would be considered to be at high risk of developing T2D, rising to 49% under the ADA defined glycaemic range (HbA1c 39–47 mmol/mol [5.7–6.4%]) [25]. It is unlikely resources will ever be available to enter this proportion of a population into an effective diabetes prevention programme [26]. The UK has 100,000 places, rising to 200,000, annually for their programme [27], a fraction of the places that would be needed to enrol the estimated 19% of the adult population, ~ 8.5 million in England [28], who will have an HbA1c ≥ 42 mmol/mol (6.0%).

We use a large UK population dataset to assess how best to target the people most at risk of developing T2D for these prevention programmes. We evaluate the relationship between HbA1c and future diabetes risk in a self-selected population cohort and determine the impact of varying test thresholds and existing clinical risk models.

## Methods

### Study population

Participant data were accessed via the Exeter 10,000/Peninsula Research Bank (EXTEND/PRB). EXTEND/PRB is an unselected population cohort of 11,074 participants, 8295 without known diabetes, recruited from the community and primary care in the South West, UK. This is an ethically approved research cohort (REC no:14/SW/1089) established to provide a resource for researchers seeking access to samples/data/potential research participants [29]. It was designed to support research into improving our understanding of common diseases and healthy ageing. Participants were recruited from GP practices through mail invites to adults registered with the practice, word of mouth, study promotion at places of work, social groups and public events and through local media. Baseline samples taken include fasting blood and urine, with associated data recorded including height, weight, blood pressure and lifestyle/medical history details. Participants are invited to provide consent for further contact and access to ongoing medical records. Follow-up blood test results were obtained from electronic laboratory records for 10,885/11,074 participants who provided consent.

**Fig. 1:**
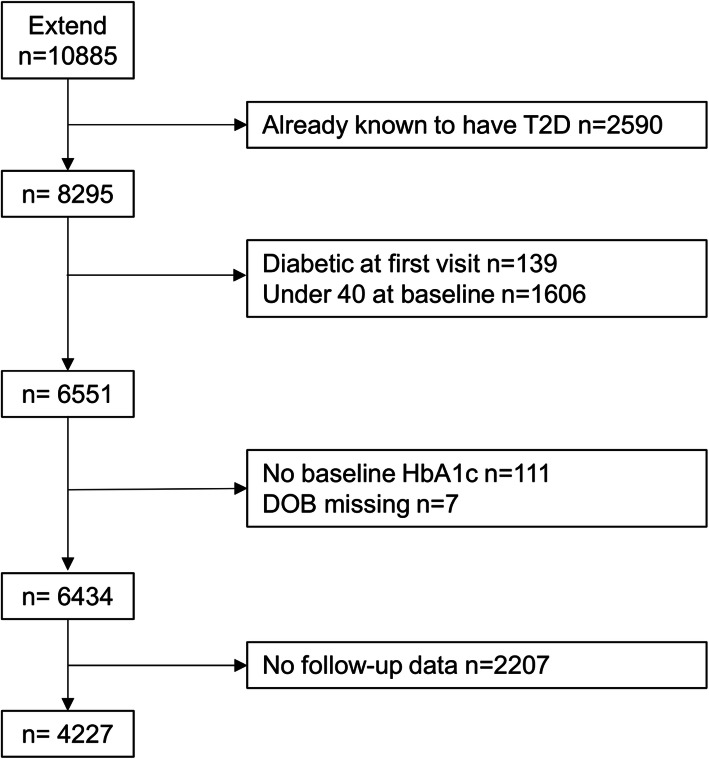
Flow chart of patients through the study

Our entry criteria based on baseline EXTEND/PRB recruitment included aged ≥ 40 years and no diagnosed diabetes (either previously diagnosed or HbA1c ≥ 48 mmol/mol [6.5%] [30]). This identified 6434 individuals (Fig. 1). An additional criterion for our study was that individuals had at least one follow-up HbA1c > 4 weeks after the baseline measurement. There were no additional values (non-follow-up) on 2207 participants (Additional file 1: Table S1). While HbA1c is not routinely measured in people without diabetes, in the UK, it is measured as part of the UK NHS Health check for those aged 40–74 years or through opportunistic screening [31]. Four thousand two hundred twenty-seven participants with follow-up data were identified for our study.

Cohort recruitment occurred between January 2010 and April 2018. Electronic laboratory records were accessed up to March 2019.

### Laboratory measurement

All included HbA1c values (baseline and follow-up) were assessed by the Academic Department of Blood Sciences at the Royal Devon and Exeter NHS Foundation Trust, using the TOSOH G8Ion Exchange High Performance Liquid Chromatography platform, calibrated to the IFCC reference preparation, intra assay CV < 2%.

### Statistical analysis

In the primary analysis, we fitted a flexible parametric survival model to estimate the absolute risk and hazard ratio (HR) of developing diabetes within 5 years given a baseline HbA1c measurement. The flexible parametric model was used, rather than Cox proportional hazards regression, as risk increased more at higher HbA1c values; this allowed for the fact that the proportional hazards assumption would not be met. For this study, we defined a diagnosis of diabetes as an HbA1c ≥ 48 mmol/mol (6.5%). For those who did not have an HbA1c ≥ 48 mmol/mol (6.5%), data were censored at either their last HbA1c (if within 5 years after baseline), or at 5 years if they had further measurements. We evaluated the 5-year risk of developing T2D using the existing HbA1c ranges for identifying those at high risk of developing diabetes; ADA (HbA1c 39–47 mmol/mol [5.7–6.4%]), IEC/UK (42–47 mmol/mol [6.0–6.4%]). We assessed the area under the receiver operating characteristic curve (AUC ROC), false positive and false negative rates, sensitivity, specificity, positive predictive value (PPV) and negative predictive value (NPV) for currently used HbA1c thresholds and assessed the impact of raising the threshold.

We also looked at the impact of combined HbA1c and clinical risk score. The Leicester Risk Score (LRS) was developed and validated on similar UK population cohorts and is recommended in NHS guidance [17, 18]. The LRS model variables are sex, age (≤ 50, 50–60, 60–70 and ≥ 70 years), a relative with diabetes (none and first-degree family history of T2D), waist circumference (< 90, 90–99, 99–110 and ≥ 110 cm), high blood pressure (previous history of or participant on antihypertensive medication) and BMI (< 25, 25–30, 30–35 and ≥ 35 kg/m^2^). The risk of developing diabetes was modelled using these covariates and an optimal cut point for high risk of developing T2D was calculated using the model [19]; a person with a LRS score ≥ 16 is considered at high risk of developing T2D. In the UK, there are three pathways to the DPP; a blood test results in the at-risk range as part of routine clinical care, a LRS ≥ 16 followed by a blood test result in the at-risk range or those who have been in the at risk group in the past [32, 33]. The use of a two-stage procedure with the clinical risk score (LRS) reduces the need for additional blood testing in those at low risk. LRS results are presented in Additional file 2.

As a DPP was introduced in the cohort area in June 2018, a sensitivity analysis with study end point June 2018 was carried out (Additional file 3).

Data were analysed using Stata v16 including the stpm2 package.

## Results

Within included participants (*n* = 4227), 3.4% (*n* = 144) of participants progressed to diabetes within a 5-year period. Mean (SD) time to progression to diabetes was 45.6 (17.9) months and mean follow-up time (SD) 28.4 (14.0) months (Table 1). Follow-up data were available for 4227 out of 6434 participants who provided baseline HbA1c. These participants had heavier BMI (mean 95% CI difference 0.8 kg/m^2^ [0.6, 1.0 kg/m^2^] kg/m^2^) and were 3.7 years (3.1, 4.3 years) older. Their baseline HbA1c was 1.7 mmol/mol (1.5, 1.9 mmol/mol), 0.15% (0.14, 0.17%), higher than those without follow-up (Table 1, Additional file 1: Table S1), suggesting those who followed up with clinical hbA1c testing had a higher risk of diabetes.

**Table 1:** Baseline characteristics of the cohort

	All participants	Participants who do not develop diabetes	Participants who develop diabetes
Age (years)	60.7 (10.8)	60.5 (10.8) *n* = 4083	65.3 (10.4) *n* = 144
Sex (% female)	62.1%	62.4% *n* = 4082	54.9% *n* = 144
BMI (kg/m^2^)	26.9 (4.5) *n* = 4223	26.8 (4.5) *n* = 4079	29.9 (5.2) *n* = 144
Weight (kg)	76.0 (15.1) *n* = 4223	75.8 (14.9) *n* = 4081	84.1 (18.1) *n* = 144
Waist (cm)	89.3 (13.1) *n* = 4217	89.0 (12.9) *n* = 4073	99.4 (13.6) *n* = 144
HbA1c (mmol/mol [%])	38.9 (3.5) (5.7 [0.3]%) *n* = 4227	38.7 (3.4) (5.7 [0.3]%) *n* = 4083	43.5 (2.6) (6.1 [0.2]%) *n* = 144
Mean systolic blood pressure (mmHg)	134.0 (18.8) *n* = 4226	133.8 (18.8) *n* = 4082	141.0 (19.3) *n* = 144
Fasting glucose (mmol/L)	5.1 (0.5) *n* = 3582	5.0 (0.5) *n* = 3465	5.6 (0.7) *n* = 117
Index of Multiple Deprivation deciles (UK population deciles)	6.9 (2.1) *n* = 4144	6.9 (2.1) *n* = 4004	6.5 (2.2) *n* = 140
Current smoker	5.6% (*n* = 235/4227)	5.4% (*n* = 219/4082)	11.1% (*n* = 16/144)
Family history of diabetes	21.7% (*n* = 918/4227)	21.4% (*n* = 841/4083)	30.6% (*n* = 44/144)
Ethnicity:			
White	98.9% (*n* = 4180/4225)	98.9% (*n* = 4037/4081)	99.3% (*n* = 143/144)
Other	1.1% (*n* = 45/4225)	1.1% (*n* = 44/4081)	0.7% (*n* = 1/144)
Follow-up time (months)^a^	45.0 (18.0) *n* = 4227	45.6 (17.9) *n* = 4083	28.4 (14.0) *n* = 144

Mean (SD) or percentage reported

^a^Shorter follow-up time in those who progressed to diabetes vs those who did not is due to censoring of time at the point of progression to T2D

### The absolute 5-year risk of developing type 2 diabetes is modest in those meeting criteria for diabetes prevention programmes

The overall absolute 5-year risk (95% CI) of developing T2D in the cohort was 4.2% (3.6,4.8%). 56% (*n* = 2358/4224) of participants were classified as high-risk based on ADA criteria (HbA1c ≥ 39 mmol/mol [5.7%]). However, the risk of progression in this group was modest: absolute 5-year risk of developing T2D 7.1% (6.1,8.2%). Using the IEC criteria, HbA1c ≥ 42 mmol/mol (6.0%), 22% (*n* = 929/4277) of the cohort was classified as ‘high-risk’, the absolute 5-year risk in this group was 14.9% (12.6, 17.2%). In those meeting both UK NICE criteria of HbA1c ≥ 42 and LRS ≥ 16, 14% (*n* = 578/4214), the 5-year risk was slightly higher at 19.0% (15.8,22.1%). In those at high clinical risk (LRS ≥16), 56% of participants identified as high-risk by US criteria had a low risk of progression: the overall 5-year risk in those with HbA1c ≥ 39 mmol/mol [5.7%] was 10.4% (8.7,12.1%) with risk 3.1% (2.0,4.0%) in the group with HbA1c 39–41 mmol/mol [5.7–5.9%].

### Raising the HbA1c threshold for entry reduces the number of people referred to prevention programmes who are unlikely to develop diabetes

**Fig. 2:**
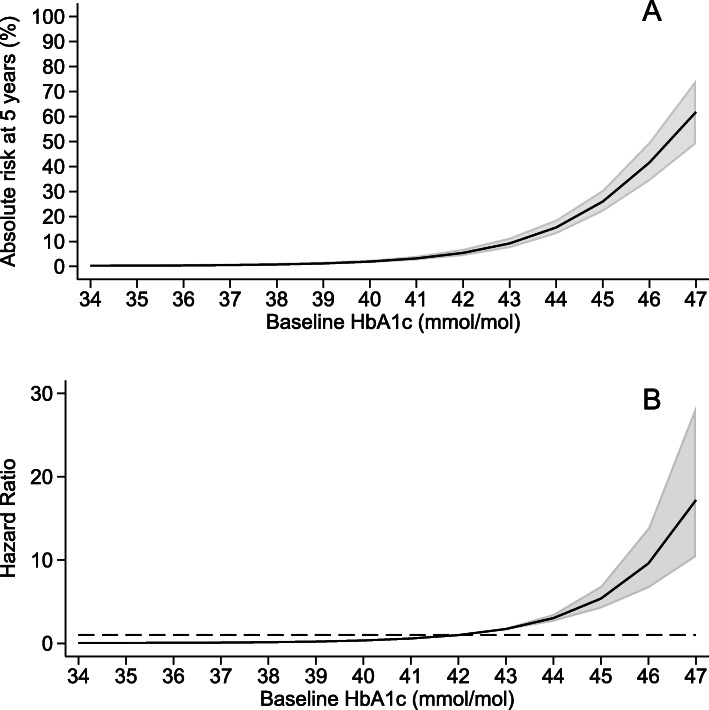
**A** Absolute 5-year risk of developing T2D (defined by HbA1c ≥ 48 mmol/mol [6.5%]) within 5 years given a baseline HbA1c modelled using a flexible parametric survival model. **B** Hazard ratio for risk of developing type T2D (defined by HbA1c ≥ 48 mmol/mol [6.5%]) within 5 years given a baseline HbA1c modelled using a flexible parametric survival model. The hazard ratio presented is relative to the cutoff value of 42 mmol/mol (6.0%). --- indicates hazard ratio of 1. HbA1c % conversion = 0.0915 × HbA1c mmol/mol + 2.15

Figure 2a (values in Additional file 4) shows absolute risk and Fig. 2b relative risks for developing HbA1c defined diabetes within 5 years of study recruitment based on a flexible parametric survival model. A Kaplan-Meier plot by HbA1c categories is shown in Additional file 5. Future risk of developing diabetes was very strongly related to HbA1c value within the current ‘high-risk’ categories. Those in the lower ranges of current ‘pre-diabetes’ criteria had a low 5-year absolute risk of developing diabetes, even where multiple other risk factors were present. For example, 5-year risk in those with the highest HbA1c values (44–47 mmol/mol [6.2–6.4%]) was 26.4% (22.0, 30.5%) compared with 2.1% (1.5,2.6%) for 39–41 mmol/mol (5.7–5.9%) and 7.0% (5.4, 8.6%) for those with an HbA1c 42–43 mmol/mol (6.0–6.1%). The hazard ratio (HR) shows that a person with an HbA1c of 44 mmol/mol (6.2%) is three times (CI 2.6–3.5) more likely to develop T2D within 5 years than someone with an HbA1c of 42 mmol (6.0%) (Fig. 2b).

**Fig. 3:**
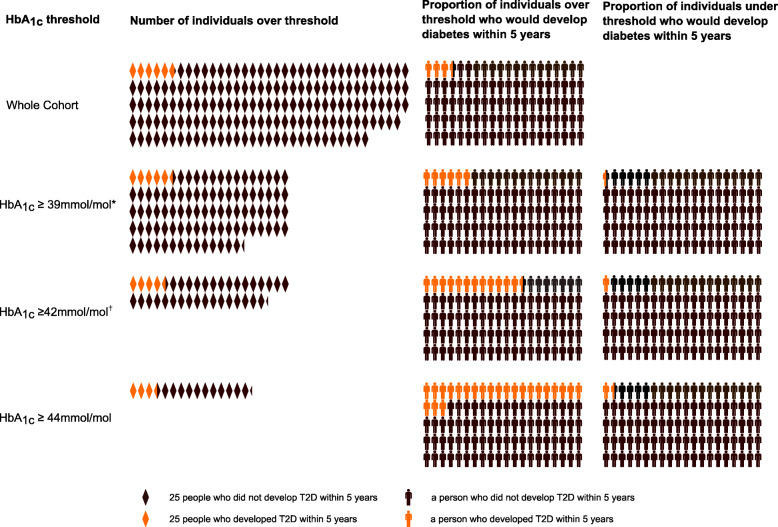
Illustration of how many people in the cohort, *n* = 4227, who are considered at high risk of developing diabetes given different HbA1c selection criteria and the proportion of those in, and excluded from, the diabetes prevention programme who would have gone on to develop diabetes; * ADA threshold, ^†^ IEC threshold

Increasing the risk threshold reduced the number of participants that would be classified as high-risk and entered into prevention programmes and increased the risk of included individuals (Table 2). Changing the US threshold of 39 mmol/mol (5.7%) to the more widely used threshold of 42 mmol/mol (6.0%) reduces the proportion classified as high-risk (and therefore eligible for intervention) by 61%. Increasing the threshold further from 42 to 44 mmol/mol (6.0% to 6.2%) reduces the proportion classified as high-risk (and therefore eligible for intervention) by a further 59%. While these changes markedly increase positive predictive value and markedly reduce false positive rates, they have very little impact on the negative predictive value, with those below the thresholds unlikely to develop diabetes within 5 years (Table 2, Fig. 3). However, higher thresholds reduce sensitivity, with 38.9% (30.9,47.3%) of those developing diabetes within 5 years missed using a threshold of > 44 mmol/mol (sensitivity 61.1% [52.6, 69.1%]). Figure 3 illustrates the impact of these thresholds on screening a population.

**Table 2:** Predictive value of HbA1c

Threshold (*T*)	*N*^a^	Percentage of participants classified as high-risk ≥ *T* (*n*)	*n* ≥ *T* who progress to diabetes	Sensitivity	*Specificity*	PPV	NPV	False positives	False negatives	AUC ROC
HbA1c ≥ 39 mmol/mol^b^ (5.7%)	4227	55.8% (*n* = 2358)	137	95.1% (90.2, 98.0)	45.6% (44.1, 47.1)	5.8% (4.9, 6.8)	99.6% (99.2, 99.8)	54.4% (52.9,55.9)	4.9% (2.0,9.8)	70.4% (68.5,72.3)
HbA1c ≥ 42 mmol/mol^c^ (6.0%)	4227	22.0% (*n* = 929)	115	79.9% (72.4, 86.1)	80.1% (78.8, 81.3)	12.4% (10.3, 14.7)	99.1% (98.7, 99.4)	19.9% (18.7,21.2)	20.1% (13.9,27.6)	80.0% (76.6, 83.3)
HbA1c ≥ 44 mmol/mol (6.2%)	4227	9.0% (*n* = 380)	88	61.1% (52.6, 69.1)	92.8% (92.0, 93.6)	23.2% (19.0, 27.7)	98.5% (98.1, 98.9)	7.1% (6.4, 8.0)	38.9% (30.9,47.3)	77.0% (73.0, 81.0)

Sensitivity, specificity, positive predictive value (PPV) and negative predictive value (NPV), false positive rate, false negative rate, AUC ROC with 95% confidence intervals for progression of our cohort to diabetes over follow-up time (mean [SD] 45.0 [18.0] months) given HbA1c thresholds

^a^*N* who progress to diabetes in cohort 144, ^b^ADA threshold, ^c^IEC threshold

### Combining assessment of a patient’s clinical risk with their HbA1c test result only very modestly improves prediction of future diabetes

The impact of combining clinic risk (using the LRS) with HbA1c testing on our model of 5-year absolute risk is shown in Additional file 2. Overall the 5-year absolute risk for an LRS ≥ 16 was 5.5% (4.5, 6.5%, *n* = 1916), slightly higher than the risk in the whole cohort, 4.2% (3.6,4.8%, n=4227), and the 5-year risk was only modestly higher in combination with high HbA1c. When assessing the accuracy of the thresholds in our cohort, using a LRS score of ≥ 16 alone had similar performance to measuring HbA1c and using a cutoff of 39 mmol/mol (5.7%), without the need for a biochemical test. However, the positive predictive value of both approaches was low: 5.9% (4.9, 7.1%) (LRS ≥ 16, NPV 98.7% [98.1, 99.1%]) and 5.8% (4.9, 6.8%) (HbA1c ≥ 39 mmol/mol [5.7%], NPV 99.6% [99.2, 99.8%]). The performance of the UK recommended two-step strategy is shown in Additional file 2 and improves the positive predictive value from the use of an HbA1c of 42 mmol/mol (6.0%) alone (from 12.4% [10.3, 14.7%] to 15.9% [13.0, 19.2%]), at the expense of a decrease in sensitivity from 79.9% (72.4, 86.1%) to 63.9% (55.5, 71.7%) but a negligible change in NPV 99.1% (98.7, 99.4%) to 98.6% (98.1, 98.9%) (Supplementary Table S3).

### Sensitivity analysis

A sensitivity analysis excluded any participants who may have been affected by the start of the UK’s DPP (Additional file 3), this reduced our cohort size to n=4105. There were no appreciable changes in the demographic characteristics of the cohort (Additional file 3: Table S4), e.g., HbA1c changed from mean (SD) 38.8 mmol/mol (3.5), 5.7% (0.3), to 38.9 mmol/mol (3.5), 5.7% (0.3). The mean follow-up time was reduced from 45.0 months to 43.3 months. A similar proportion of participants progressed to T2D, 3.2% (*n* = 130/4105), and the absolute risk of progression within 5 years was similar; at a threshold of 39 mmol/mol (5.7%) the risk was 4.2% (3.6,4.8%), 7.1% (6.1,8.2%) and 14.9% (12.6,17.2%) for thresholds of 42 and 44 mmol/mol (6.0% and 6.2%), respectively.

## Discussion

We have shown in our UK population cohort that current guidance for using HbA1c to identify those at risk of future diabetes classifies a large proportion of the population as high-risk (often termed pre-diabetes). Overall, for many individuals, the absolute risk of developing diabetes over a 5-year period is very low, particularly for those at the lower end of the ‘high-risk’ HbA1c range. A potential approach to target those most at risk would be to raise the inclusion threshold, which would markedly reduce the numbers at low risk undergoing intervention, at the expense of loss of sensitivity, with a proportion of who progress to T2D missed by a move to higher thresholds. Raising HbA1c thresholds from 39 mmol/mol to 42 mmol/mol (5.7–6.0%; ADA) or from 42 mmol/mol to 44 mmol/mol (6.0–6.2%; IEC and UK) would avoid classifying a large proportion of the population who have low absolute risk as having a pre-disease state, and could potentially increase cost-effectiveness of targeted prevention clinical programs and research studies where resources are insufficient to target the entire at-risk population.

Longitudinal cohort studies mostly focus on FPG or IGT, with only *n* = 6/70 studies in a meta-analysis of progression to T2D using HbA1c to define pre-diabetes [22]. A UK cohort study of 5735 participants had 1.3% progressing to T2D over 3 years and showed an increased risk of developing diabetes with increasing HbA1c, 7 times greater risk of developing diabetes in the group with HbA1c 42–46 mmol/mol (6.0–6.4%) vs < 31 mmol/mol (5.0%) [34]. Most studies used the ADA or IEC HbA1c or IFG ranges to identify individuals at high risk of developing diabetes without additional focus on the impact of raising the threshold higher than 42 mmol/mol (6.0%). Three studies outside the UK had similar duration, 5 years in the Danish (Inter99, *n* = 4930) and Australian (AusDiab, *n* = 6012) studies and 6 years in the French DESIR (*n* = 3784) study [35]. These studies found similar incidences of diabetes to our study, 2.3–3.1%; however, modestly lower sensitivities were reported (65–78% using a threshold of 39 mmol/mol [5.7%] and 38–45% at a threshold of 42 mmol/mol [6.0%]). The increase in PPV observed with increasing HbA1c thresholds was consistent with our findings, 5.9–11% at ≥ 39 mmol/mol (5.7%), 13–28% for ≥ 42 mmol/mol (6.0%) and 27–44% for ≥ 46 mmol/mol (6.4%).

Other work has concluded that prevention programmes should target those at the highest risk. In a recent study of a UK prevention programme, Smith et al. [36] conclude that to maximise the effectiveness programme eligibility criteria should be adjusted to target more high-risk individuals. Zhuo et al. found lowering fasting glucose thresholds lowered the cost-effectiveness of programmes [37]. A cost-effectiveness analysis by Thomas et al. suggests that the best value, and best health benefits, for a prevention programme, would be to prioritise obese (BMI ≥ 30 kg/m^2^) individuals, those with the highest HbA1c in the pre-diabetes range and aged 40–74 [38]. A higher HbA1c threshold for entry to a prevention programme targets those at the highest risk.

The strengths of this study include the use of a large unselected population cohort and the assessment of clinical risk using models and thresholds previously validated and routinely used in this region. In particular, this study adds to the understanding of the predictive capacity of HbA1c in progression to T2D, identified as an area requiring further research [17, 22]. A key limitation of our study is that follow-up HbA1c relied on clinical testing and was therefore not available in all study participants. This may have introduced bias as factors that influence the risk of T2D may also influence whether a clinician screens for diabetes. This is consistent with those who provided baseline data only in our cohort who were on average 3.7 years younger, 1.9 kg lighter and had 1.7 mmol/mol (0.15%) lower HbA1c, than our cohort. This suggests higher-risk patients were more likely to be followed up and therefore included in our cohort, and we may therefore overestimate future diabetes risk. However, the characteristics of included participants are broadly consistent with previous UK population studies of older adults reporting HbA1c, for example, the Norfolk (*n* = 3921), Leicester (*n* = 6390) and ELSA (*n* = 5262) studies reported mean HbA1c of 36, 39 and 36 mmol/mol (5.4, 5.7, 5.4%), BMI 30.1, 28.1 and 27.5 km/m^2^, and age 58.8, 57.3 and 65.8 years, respectively [18, 39, 40].

A further limitation is that our cohort contains limited numbers of people of non-white ethnicity (consistent with the population of this region) and predictive value of HbA1c and performance of HbA1c thresholds may be different in other ethnicities [6, 22, 25, 41]. For example, Mostafa et al. showed that equivalent HbA1c thresholds differed in a study on white Europeans and south Asians, 5.7% and 6.0%, respectively [25]. The progression rates to diabetes based on an HbA1c 6.0–6.4% have been shown to differ across ethnicities: incidence rate ratios (95% CI) 0.21 (0.02,2.03) in Europe and 0.30 (0.02, 4.96) in the Americas relative to Asia [22]. Further work should replicate our study in cohorts with more diverse ethnic profiles.

Lastly, we have used HbA1c for both risk prediction and diagnosis. We may have seen a different relationship had glucose-based measures been used to define diabetes, with potentially lower sensitivity had multiple measures (such as OGTT) been used to define incident diabetes. However, this closely reflects clinical practice—in the UK, T2D is now diagnosed principally by HbA1c testing, and almost all of those diagnosed will have had an earlier HbA1c test. Fasting glucose is tested rarely and glucose tolerance tests are used only in the setting of detecting gestational diabetes.

Our findings have potential implications for clinical practice. HbA1c is commonly used in practice to determine if a person should be identified as high risk (often termed ‘pre-diabetes’) and referred to a DPP, and, with current thresholds, a high proportion (up to 54% with ADA criteria) would be eligible, with low 5-year risk of diabetes for those with HbA1c in the lower part of ‘pre-diabetes’ risk range. Currently, those in the pre-diabetes range are given equal weighting for an invitation to a prevention programme. Where resources or capacity are constrained, higher thresholds could be used to refer to these programs, with those with lower HbA1c potentially offered monitoring of HbA1c with referral only if HbA1c progresses to a higher risk range. However, this would be balanced with a trade-off in cases missed. Ultimately, where resources and capacity allow, the optimal choice of threshold should be based on detailed cost-effectiveness analysis, taking into account multiple factors including the costs of preventable cases that may be missed with higher test thresholds, program costs and the potential negative impact of ‘false positive’ results.

## Conclusion

In a UK population, a large proportion of people are identified as high-risk using current thresholds, with modest 5-year risk of diabetes. Increasing the risk threshold markedly reduces the number of people that would be classified as high-risk and entered into prevention programmes, allowing limited intervention opportunities to be focused on those most likely to develop T2D.

## Supplementary Information

**Additional file 1.** Characteristics of those who provided baseline data only.

**Additional file 2.** Leicester Risk Score.

**Additional file 3.** Sensitivity analysis excluding data after the launch of the UK diabetes prevention programme.

**Additional file 4.** Absolute 5 year risk of developing Type 2 diabetes given baseline HbA1c.

**Additional file 5.** Kaplan-Meier survival plot by HbA1c category.

## Data Availability

The authors do not have permission to share these data. Access to the EXTEND/PRB resource is governed and approved by the Peninsula Research Bank Steering Committee. Requests for access to original data should be in the form of a formal application the Peninsula Research Bank details can be found at: https://exetercrfnihr.org/about/exeter-10000-prb/.

## Abbreviations

ADA: American Diabetes Association
AUC ROC: Area under the receiver operating characteristic curve
CI: Confidence interval
DPP: Diabetes prevention programme
EXTEND: Exeter 10,000 cohort
FPG: Fasting plasma glucose
IEC: International Expert Committee
IQR: Inter quartile range
LRS: Leicester Risk Score
NHS: National Health Service
NICE: National Institute for Health Care Excellence
NPV: Negative predictive value
OGTT: Oral glucose tolerance test
pp: Per person
PPV: Positive predictive value
PRB: Peninsula Research Bank
T2D: Type 2 diabetes
UK: United Kingdom
WHO: World Health Organization
