# Supplementary material for: Choice of HbA1c threshold for identifying individuals at high risk of type 2 diabetes and implications for diabetes prevention programmes: a cohort study
Source: BMC Med. 2021 Aug 20;19:184. doi: 10.1186/s12916-021-02054-w (PMC8377980; doi:10.1186/s12916-021-02054-w)
Supplement: Supplementary file 1 — Additional file 1. Characteristics of those who provided baseline data only. [file 12916_2021_2054_MOESM1_ESM.docx]

**Additional File 1: Table S1 Characteristics of those who provided baseline data only**

|  | Lost to follow-up | All participants | Difference (Mean 95% CI) |
| --- | --- | --- | --- |
| Age (years) | 56.0 (47.0-66.0) | 60.7 (10.8) | -3.7 (-4.3,-3.1) |
| Sex (% Female) | 64.8% | 62.1% | 2.7% (0.2,5.2) |
| BMI (kg/m^2^) | 25.5 (23.1-28.4) | 26.9 (4.5) n=4223 | -0.8 (-1.0,-0.6) |
| Weight (kg) | 72.1 (63.4-83.4) | 76.0 (15.1) n=4223 | -1.9 (-2.7, -1.1) |
| Waist (cm) | 86.0 (74.0-95.2) (n=2204) | 89.3 (13.1) n=4217 | -2.2 ( -2.8,-1.5) |
| HbA1c (mmol/mol; %) | 37 (35-39) (5.5 [5.4-5.7]%) | 38.9 (3.5)  (5.7 [0.3]%) n=4227 | -1.7 (-1.9,-1.5) -0.15% (-0.17,-0.14) |
| Mean systolic blood pressure (mmHg) | 128 (117-140) | 134.0 (18.8) n=4226 | -4.1 (-5.1,-3.1) |
| Fasting glucose (mmol/L) | 4.9 (4.6-5.2) (n=1850) | 5.1 (0.5) n=3582 | -0.1 (-0.1,-0.1) |
| Index of Multiple Deprivation deciles | 7 (5-8) n=2166 | 6.9 (2.1) n=4144 | -0.3 (-0.4,-0.2) |
| Current smoker | 5.2% (n=109/2103) | 5.6% (n=235/4227) | -0.4% (-1.6,0.1) |
| Family history of diabetes | 13.8% (n=304/2207) | 21.7% (n=918/4227) | -8.4% (-9.2, -5.5) |
| Ethnicity: White  Other | 98.2% (n=2166/2206)  1.8% (n=40/2207) | 98.9% (n=4180/4225)  1.1% (n=45/4225) | 0.7% (0.1, 1.4) |
| Year of recruitment | 2010 12.6% 2011 14.6% 2012 7.8% 2013 4.7% 2014 6.6% 2015 11.8% 2016 16.3% 2017 13.0% 2018 12.6% | 2010 21.0% 2011 26.0% 2012 14.4% 2013 10.0% 2014 8.0% 2015 9.0% 2016 8.3% 2017 3.0% 2018 0.4% | 1.8 (1.7,2.0)* |

Mean (SD) or percentage. n=2207 (lost to follow up) or n=4227 (follow up data available) unless otherwise stated. *Differences in average year of recruitment
