# Supplementary material for: Choice of HbA1c threshold for identifying individuals at high risk of type 2 diabetes and implications for diabetes prevention programmes: a cohort study
Source: BMC Med. 2021 Aug 20;19:184. doi: 10.1186/s12916-021-02054-w (PMC8377980; doi:10.1186/s12916-021-02054-w)
Supplement: Supplementary file 2 — Additional file 2. Leicester Risk Score. [file 12916_2021_2054_MOESM2_ESM.docx]

**Additional File 2: Leicester Risk Score (LRS)**

5.9% (n=113/1916) who had LRS≥16 developed diabetes within 5 years.

In those with a LRS≥16, the overall absolute 5 year risk of developing diabetes was 5.5% (4.5,6.5)%. Within those with LRS≥16 the absolute 5 year risk for a given HbA1c threshold; 10.4% (8.7,2.1%) for a 39mmol/mol threshold, 19.0% (15.8,22.1%) for a 42mmol/mol threshold and 32.5% (26.8,37.8%) for a 44mmol/mol threshold.

**Supplementary Figure S1**: Absolute risk of developing Type 2 diabetes for LRS.
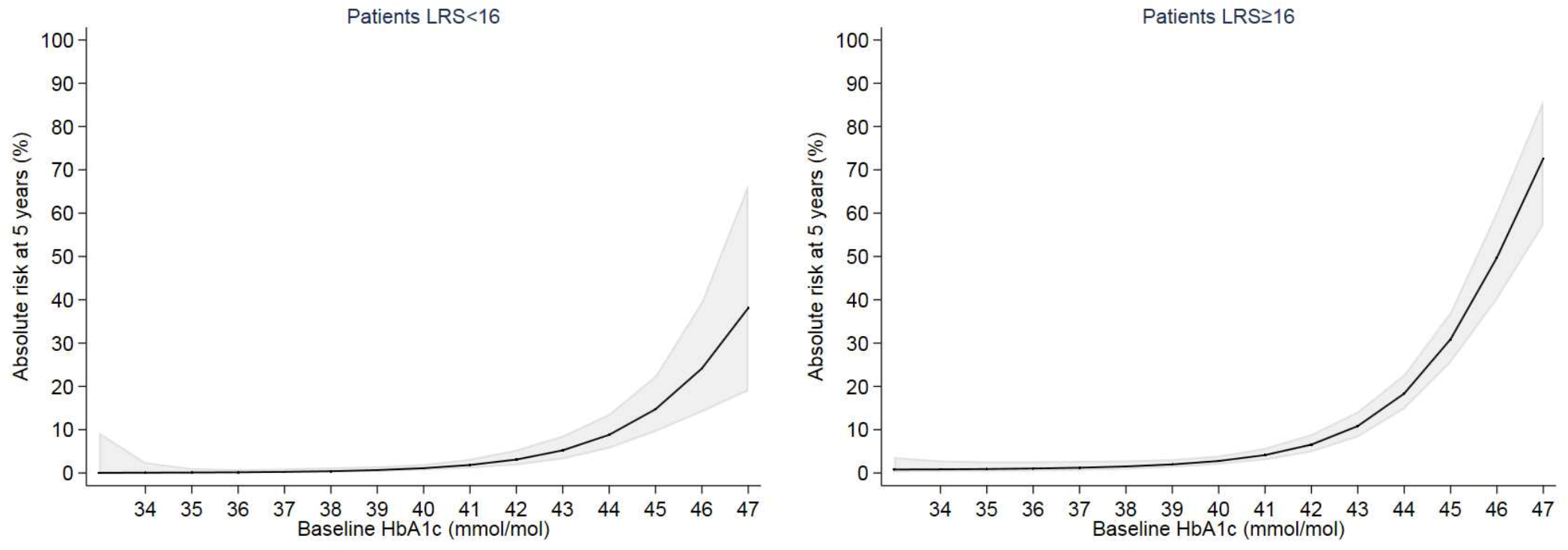


**Supplementary Figure S2: Hazard ratio for LRS categories.**


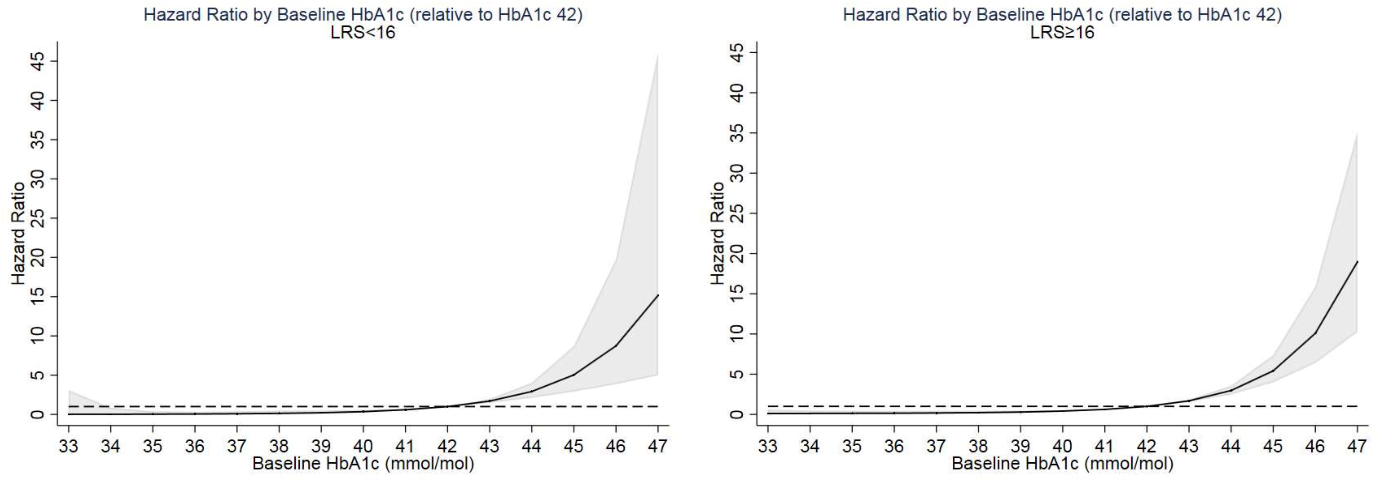


**Supplementary Table S2**: Absolute 5 year risk of developing Type 2 diabetes given baseline HbA1c % (mmol/mol). * ADA threshold, ^†^IEC threshold, UK threshold HbA1c 6.2% (42mmol/mol) and LRS≥16.

| HbA1c % (mmol/mol) | All Patients | | LRS<16 | | LRS≥16 | |
| --- | --- | --- | --- | --- | --- | --- |
|  | 5yr Abs Risk (95% CI) | N | 5yr Abs Risk (95% CI) | N | 5yr Abs Risk (95% CI) | N |
| Overall | 4.2% (3.6,4.8) | 4227 | 2.4% (1.6,3.2) | 2298 | 5.5% (4.5,6.5) | 1916 |
| ≤36mmol/mol (5.4%) | 0.3% (0.0,0.7) | 959 | 0.1% (0.0,0.3) | 656 | 0.9% (0.0,2.0) | 300 |
| 37mmol/mol (5.5%) | 0.5% (0.2,0.9) | 427 | 0.3% (0.0,0.6) | 276 | 1.1% (0.2,2.0) | 147 |
| 38mmol/mol (5.6%) | 0.8% (0.4,1.2) | 484 | 0.4% (0.0,0.9) | 275 | 1.5% (0.5,2.4) | 209 |
| 39*mmol/mol (5.7%) | 1.2% (0.7,1.7) | 513 | 0.7% (0.1,1.3) | 289 | 2.0% (1.0,2.9) | 221 |
| 40mmol/mol (5.8%) | 2.0% (1.4,2.5) | 484 | 1.2% (0.5,1.8) | 249 | 2.8% (1.9,3.8) | 235 |
| 41mmol/mol (5.9%) | 3.2% (2.4,4.1) | 432 | 1.9% (0.9,2.9) | 205 | 4.3% (3.0,5.7) | 226 |
| 42^†^mmol/mol (6.0%) | 5.4% (4.1,6.8) | 320 | 3.2% (1.5,4.8) | 136 | 6.9% (4.9,8.9) | 181 |
| 43mmol/mol (6.1%) | 9.2% (7.1,11.2) | 229 | 5.3% (2.7,7.8) | 79 | 11.4% (8.5,14.3) | 150 |
| 44mmol/mol (6.2%) | 15.6% (12.7,18.4) | 173 | 8.8% (5.0,12.5) | 66 | 19.1% (15.2,22.9) | 107 |
| 45mmol/mol (6.3%) | 26.0% (21.5,30.2) | 115 | 14.6% (8.4,20.4) | 44 | 31.7% (25.9,37.1) | 71 |
| 46-47mmol/mol (6.4-6.5%) | 47.3% (37.2,55.7) | 92 | 32.0% (10.8,48.1) | 23 | 54.1% (42.2,63.5) | 69 |

**Supplementary Table S3**: Effect of thresholds with high clinical risk score with 95% confidence intervals.

| Threshold (T) | N^*^ | Percentage of participants classified as high risk ≥ T (n) | n ≥ T who progress to diabetes | Sensitivity | Specificity | | PPV | NPV | False positives | False negatives | AUC ROC |
| --- | --- | --- | --- | --- | --- | --- | --- | --- | --- | --- | --- |
| *High Clinical Risk* | | | | |  |  |  |  |  |  |  |
| LRS≥16 | 4214 | 45.5% (n=1916) | 113 | 78.5% (70.9, 84.9) | 55.7% (54.2, 57.2) | | 5.9% (4.9, 7.1) | 98.7% (98.1, 99.1) | 44.3% (40.8, 45.8) | 21.5% (15.1,29.1) | 67.1% (63.6, 70.5) |
| *Combined Clinical Risk and HbA1c* | | | | | | | | | | | |
| LRS≥16 & HbA1c≥39mmol/mol (5.7%) | 4214 | 29.9% (n=1260) | 108 | 75.0% (67.1, 81.8) | 71.7% (70.3, 73.1) | | 8.6% (7.1, 10.3) | 98.8% (98.3, 99.1) | 28.3% (26.9, 29.67) | 25.0% (18.2,32.9) | 73.3% (69.7, 77.0) |
| LRS≥16 & HbA1c≥42mmol/mol (6.0%) | 4214 | 13.7% (n=578) | 92 | 63.9% (55.5, 71.7) | 88.1% (87.0, 89.0) | | 15.9% (13.0, 19.2) | 98.6% (98.1, 98.9) | 11.9% (11.0, 13.0) | 36.1% (28.3,44.5) | 76.0% (72.0, 79.9) |
| LRS≥16 & HbA1c≥44mmol/mol (6.2%) | 4214 | 5.9% (n=247) | 73 | 50.7% (42.2, 59.1) | 95.7% (95.1, 96.3) | | 29.6% (23.9, 35.7) | 98.2% (97.7, 98.6) | 4.3% (3.7, 4.9) | 49.3% (40.9,57.8) | 73.2% (69.1, 77.3) |
