# Supplementary material for: Choice of HbA1c threshold for identifying individuals at high risk of type 2 diabetes and implications for diabetes prevention programmes: a cohort study
Source: BMC Med. 2021 Aug 20;19:184. doi: 10.1186/s12916-021-02054-w (PMC8377980; doi:10.1186/s12916-021-02054-w)

**Additional File 3: Sensitivity analysis excluding data after the launch of the UK diabetes prevention programme.**

Some of our cohort may have been referred to the UKs DPP which launched in June 2018. Main analyses were repeated up to 01/07/2018.

**Additional File 3 Table S4: Baseline characteristics of the cohort, Mean (SD) or percentage reported**

| Age (years) | 60.6 (10.8) n=4105 |
| --- | --- |
| BMI (kg/m^2^) | 26.9 (4.5) n=4101 |
| Weight (kg) | 76.1 (15.0) n=4101 |
| Waist (cm) | 89.4 (13.1) n=4095 |
| HbA1c (% [mmol/mol]) | 38.8 (3.5)  (5.7 [0.3]) n=4105 |
| Mean systolic blood pressure (mmHg) | 134.1 (18.9) n=4104 |
| Fasting glucose (mmol/L) | 5.1 (0.5) (n=3479) |
| Index of Multiple Deprivation (UK population deciles) | 6.9 (2.1) n=4024 |
| Current smoker | 5.5% (n=227/4105) |
| Family history of diabetes | 21.8% (n=896/4105) |
| Ethnicity: White  Other | 99.0% (n=4062/4103)  1.0% (n=41/4103) |
| Follow up time (months) | 43.3 (18.7) |

n=4105 unless otherwise stated.

3.2% (n=130/4105) developed diabetes within 5 years. Absolute 5 year risk of developing diabetes modelled using a flexible parametric survival model: Overall 4.2% (3.5,4.8%) Threshold of 39mmol/mol 7.1% (6.0,8.3%), 42 mmol/mol 14.7% (12.3,17.1%), 44 mmol/mol 26.1% (21.5,30.4%), LRS≥16 and HbA1c 42mmol/mol 18.6% (15.3,21.8%).

**Supplementary Table S5:** Predictive value of HbA1c: AUC, false positive, false negative, sensitivity, specificity, positive predictive value (PPV) and negative Predictive value (NPV) with 95% confidence intervals for progression of our cohort to diabetes over follow up time (mean [sd] 43.3 [18.7] months) given HbA1c and LRS score thresholds. *N who progress to diabetes in cohort 130, ^†^ADA threshold, ^‡^ IEC threshold, ^§^UK NICE guidelines threshold

| Threshold (T) | N^*^ | Percentage of participants classified as high risk ≥ T (n) | n ≥ T who progress to diabetes | AUC | False positives | False negatives | PPV | NPV | Sensitivity | Specificity |
| --- | --- | --- | --- | --- | --- | --- | --- | --- | --- | --- |
| *HbA1c Threshold (n=130 develop diabetes)* | | | | | | | | | | |
| HbA1c≥39 mmol/mol^†^ (5.7%) | 4105 | 55.4% (n=2274) | 123 | 70.3% (68.2,72.3) | 54.1% (52.5,55.7) | 5.4%  (2.2, 10.8) | 5.4%  (4.5, 6.4) | 99.6% (99.2, 99.8) | 94.6% (89.2, 97.8) | 45.9% (44.3, 47.5) |
| HbA1c≥42 mmol/mol^‡^ (6.0%) | 4105 | 22.3% (n=912) | 104 | 79.8%  (76.3, 83.3) | 20.3% (19.1,21.6) | 20.0%  (13.5, 27.9) | 11.4%  (9.4, 13.6) | 99.2% (98.8, 99.5) | 80.0% (72.1, 86.5) | 79.7% (78.4, 80.9) |
| HbA1c≥44 mmol/mol (6.2%) | 4105 | 9.1% (n=373) | 80 | 77.1%  (72.9, 81.3) | 7.1%  (6.6,8.2) | 38.5%  (30.1, 47.4) | 21.4% (17.4, 26.0) | 98.7% (98.2, 99.0) | 61.5% (52.6, 69.9) | 92.6% (91.8, 93.4) |
| *Clinical Risk Score Threshold (n=130 develop diabetes)* | | | | | | | | | | |
| LRS≥16 | 4093 | 45.6% (n=1867) | 101 | 66.6%  (62.9, 70.2) | 44.6%  (43.0, 46.1) | 22.3%  (15.5, 30.4) | 5.4%  (4.4, 6.5) | 98.7% (98.1, 99.1) | 78.3% (69.6, 84.5) | 55.4% (53.9, 57.0) |
| *Combined Clinical Risk and HbA1c (n=130 develop diabetes)* | | | | | | | | | | |
| LRS≥16 &  HbA1c≥ 39 mmol/mol (5.7%) | 4093 | 29.9% (n=1225) | 96 | 72.7%  (68.8, 76.5) | 28.5%  (27.1, 29.9) | 26.2%  (18.8, 34.6) | 7.8%  (6.4, 9.5) | 98.8% (98.3, 99.2) | 73.8% (65.4, 81.2) | 71.5% (70.1, 72.9) |
| LRS≥16 & HbA1c≥ 42mol/mol^§^ (6.0%) | 4093 | 14.0% (n=571) | 82 | 75.4%  (71.2, 79.6) | 12.3%  (11.3, 13.4) | 36.9%  (28.6, 45.8) | 14.4% (11.6, 17.5) | 98.6% (98.2, 99.0) | 63.6% (54.2, 71.4) | 87.7% (86.6, 88.7) |
| LRS≥16 & HbA1c≥ 44mmol/mol (6.2%) | 4093 | 6.0% (n=244) | 66 | 73.1%  (68.8, 77.5) | 4.5% (3.9, 5.2) | 49.2%  (40.4, 58.1) | 27.0% (51.6, 33.1) | 98.3% (97.9, 98.7) | 50.8% (41.9, 59.6) | 95.5% (94.8, 96.1) |

**Supplementary Figure S3:** Absolute 5 year risk of developing Type 2 diabetes (defined by HbA1c ≥48mmol/mol) within 5 years given a baseline HbA1c; modelled using a flexible parametric survival model. Excluding data after June 2018. HbA1c % conversion = 0.0915 x HbA1c mmol/mol + 2.15


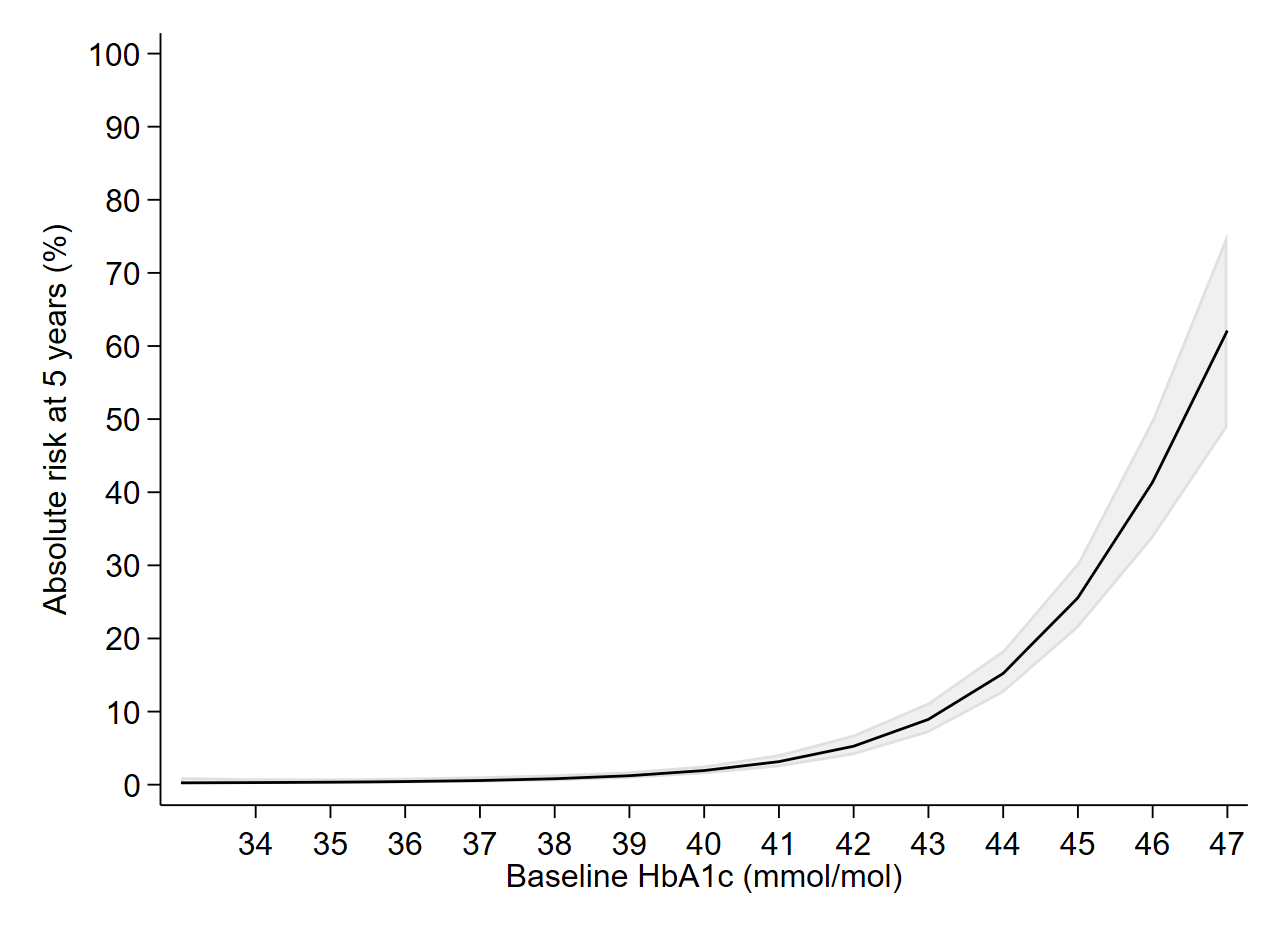


**Supplementary Figure S4:** Hazard ratio for risk of developing type 2 diabetes (defined by HbA1c ≥48mmol/mol) within 5 years given a baseline HbA1c; modelled using a flexible parametric survival model. Hazard ratio presented are relative to the cut off value of 42mmol/mol. --- indicates hazard ratio of 1. Excluding data after June 2018. HbA1c % conversion = 0.0915 x HbA1c mmol/mol + 2.15


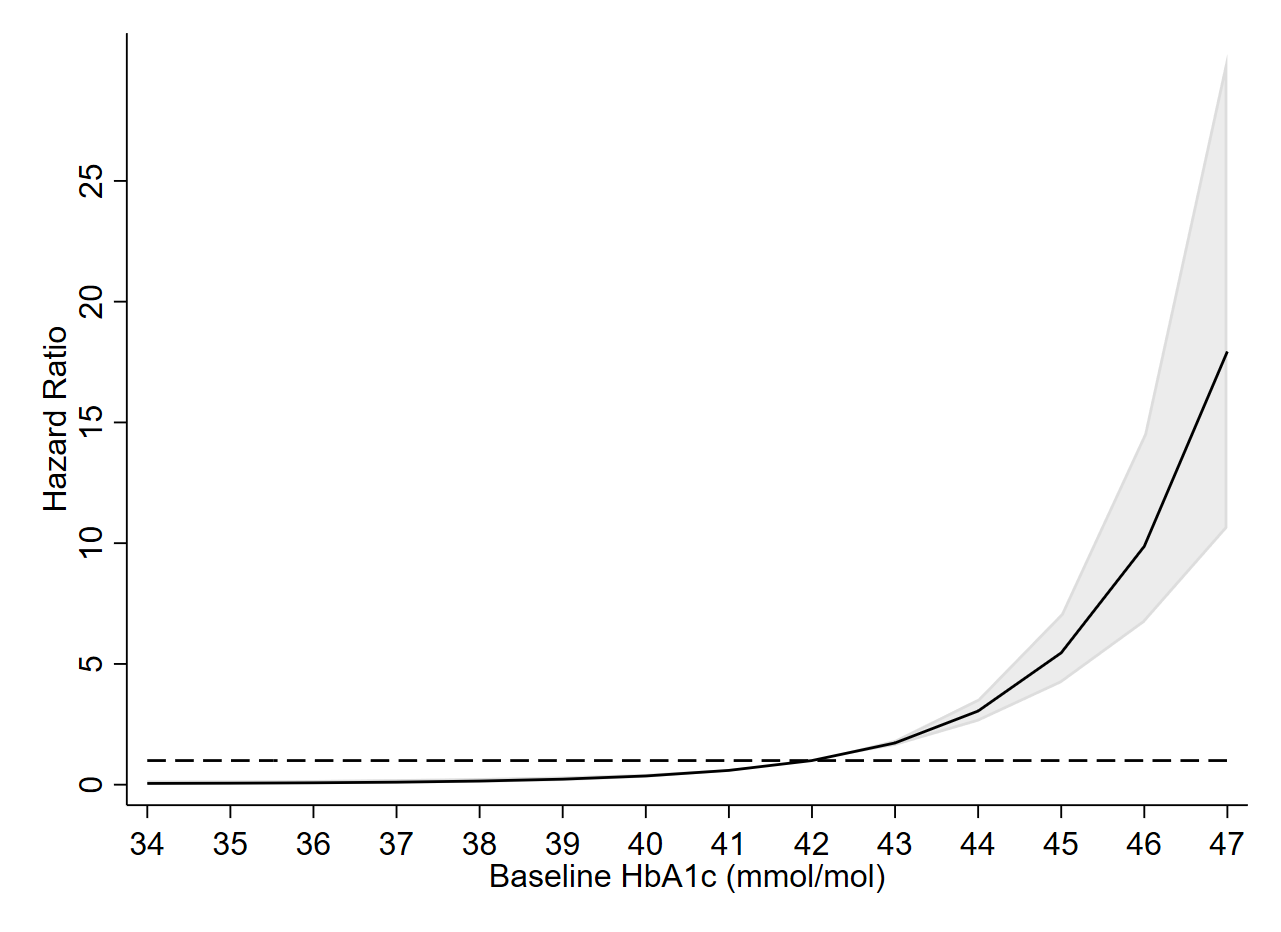

Supplement: Supplementary file 3 — Additional file 3. Sensitivity analysis excluding data after the launch of the UK diabetes prevention programme. [file 12916_2021_2054_MOESM3_ESM.docx]
