# Supplementary material for: Choice of HbA1c threshold for identifying individuals at high risk of type 2 diabetes and implications for diabetes prevention programmes: a cohort study
Source: BMC Med. 2021 Aug 20;19:184. doi: 10.1186/s12916-021-02054-w (PMC8377980; doi:10.1186/s12916-021-02054-w)
Supplement: Supplementary file 4 — Additional file 4. Absolute 5 year risk of developing Type 2 diabetes given baseline HbA1c. [file 12916_2021_2054_MOESM4_ESM.docx]

**Additional File 4: Table S6**  Absolute 5 year risk of developing Type 2 diabetes given baseline HbA1c

| HbA1c % (mmol/mol) | All Patients | | LRS<16 | | LRS≥16 | |
| --- | --- | --- | --- | --- | --- | --- |
|  | 5yr Abs Risk (95% CI) | N | 5yr Abs Risk (95% CI) | N | 5yr Abs Risk (95% CI) | N |
| Overall | 4.2% (3.6,4.8) | 4227 | 2.4% (1.6,3.2) | 1916 | 5.5% (4.5,6.5) | 2298 |
| ≤5.4 (36) | 0.3% (0.0,0.7) | 959 | 0.1% (0.0,0.3) | 656 | 0.9% (0.0,2.0) | 300 |
| 5.5 (37) | 0.5% (0.2,0.9) | 427 | 0.3% (0.0,0.6) | 276 | 1.1% (0.2,2.0) | 147 |
| 5.6 (38) | 0.8% (0.4,1.2) | 484 | 0.4% (0.0,0.9) | 275 | 1.5% (0.5,2.4) | 195 |
| 5.7* (39) | 1.2% (0.7,1.7) | 513 | 0.7% (0.1,1.3) | 289 | 2.0% (1.0,2.9) | 212 |
| 5.8 (40) | 2.0% (1.4,2.5) | 484 | 1.2% (0.5,1.8) | 249 | 2.8% (1.9,3.8) | 225 |
| 5.9 (41) | 3.2% (2.4,4.1) | 432 | 1.9% (0.9,2.9) | 205 | 4.3% (3.0,5.7) | 216 |
| 6.0^†^ (42) | 5.4% (4.1,6.8) | 320 | 3.2% (1.5,4.8) | 136 | 6.9% (4.9,8.9) | 180 |
| 6.1 (43) | 9.2% (7.1,11.2) | 229 | 5.3% (2.7,7.8) | 79 | 11.4% (8.5,14.3) | 146 |
| 6.2 (44) | 15.6% (12.7,18.4) | 173 | 8.8% (5.0,12.5) | 66 | 19.1% (15.2,22.9) | 107 |
| 6.3 (45) | 26.0% (21.5,30.2) | 115 | 14.6% (8.4,20.4) | 44 | 31.7% (25.9,37.1) | 69 |
| 6.4 (46-47) | 47.3% (37.2,55.7) | 92 | 32.0% (10.8,48.1) | 23 | 54.1% (42.2,63.5) | 68 |

% (mmol/mol). * ADA threshold, ^†^IEC threshold, UK threshold HbA1c 6.2% (42mmol/mol) and LRS≥16.
