# Supplementary figures and images for: Choice of HbA1c threshold for identifying individuals at high risk of type 2 diabetes and implications for diabetes prevention programmes: a cohort study
Source: BMC Med. 2021 Aug 20;19:184. doi: 10.1186/s12916-021-02054-w (PMC8377980; doi:10.1186/s12916-021-02054-w)

**Additional File 5: Figure S5 Kaplan-Meier survival plot by HbA1c category**


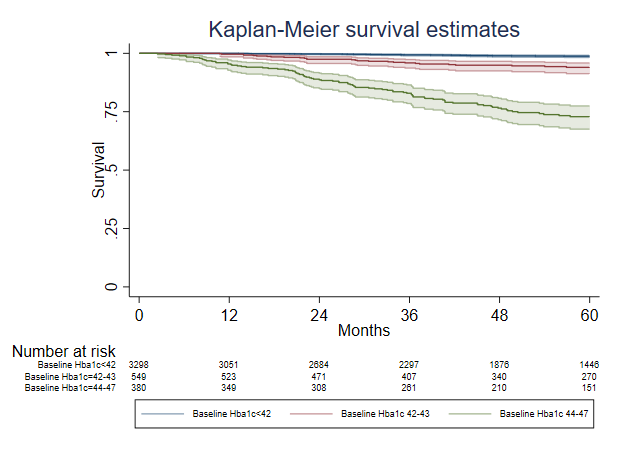

Supplement: Supplementary file 5 — Additional file 5. Kaplan-Meier survival plot by HbA1c category. [file 12916_2021_2054_MOESM5_ESM.docx]
